# The educator as facilitator of trust in the nursing education environment

**DOI:** 10.1371/journal.pone.0266240

**Published:** 2022-08-22

**Authors:** Ellie Catharina van Dyk, Gisela Hildegard van Rensburg, Elsie Sophia Janse van Rensburg

**Affiliations:** Department of Health Studies, University of South Africa, Pretoria, Gauteng, South Africa; Universitat Luzern, SWITZERLAND

## Abstract

**Background:**

Trust and trusting are inclinations to believe in the honesty and sincerity of a person. Trust and trusting relationships in nursing education form the foundation of safety, acknowledging vulnerabilities and allowing reciprocal expectations and beliefs to rely on each other during the teaching and learning process. The purpose of this article is to explore the views of nurse educators in facilitating trust and trusting relationships during teaching and learning.

**Methods and findings:**

This article focuses on the role of educators in facilitating the development of trust. A qualitative approach with a grounded theory design was used. The fourteen nurse educators included to address the first objective of the study, were purposively sampled, and the requisite data was collected by conducting individual in-depth face-to-face interviews. Initial, focus and theoretical coding were used to analyse the data until saturation was evident. The following themes emerged: competencies of the educator; setting of standards and the maintaining of consistency; and professional credibility and the value of trusting relationships. The outcome of the findings indicated that the educator plays a key role in establishing trust in nursing education. The study did not include professional nurses in practice as role-players in the nursing education environment. This could be regarded as a limitation, as the professional nurse in practice also has a teaching role, thus, indirectly, forming part of the nursing education environment. A recommendation was made that further research on trust should include professional nurses.

**Conclusions:**

The study highlights that trust is dependent on, and facilitated by, professional conduct, ethical behaviour, competencies, knowledge and skills of the role-players in the teaching and learning environment. The recommendations made have a bearing on developing trust and trusting relationships in nursing education.

## Introduction

Trust is vital and part of all relationships in the teaching and learning context [1], with it being described as the cornerstone of any human relationship that is important throughout a person’s life. Basic trust develops from birth, with trust being the first psychosocial development phase of a human being [2]. Erik Erikson (1959) in Van Vuren [2], states that trust is the first stage in psychosocial development since birth, and, with the synthesis of basic trust and mistrust, hope develops, which is a characteristic that is of great importance throughout life [3]. Castelfranchi and Falcone [4] summarise the trust concept in terms of five different aspects. First, trust is associated with an expectation, a belief, a willingness and an attitude. Second, those who trust are the individuals with the characteristics, competencies and capacities to trust. Third, actions and behaviours are important for the development and main tenance of trusting relationships. Fourth, there should be results and outputs that are predictable and favourable in terms of the specific situation. Finally, risks and uncertainties exist, in terms of which people put themselves in a vulnerable position [4]. This article reflects on the educator’s facilitation of trust by means of demonstrating a professional attitude and competencies that display the actions of ethical conduct. In short, the educator creates a safe platform for student support and development.

Trust in the self and in one’s own knowledge is considered important for students, in terms of them building up new ideas and expanding their knowledge [5]. A learning environment in which values are constantly shaping the learning process is marked by an improvement in students’ learning, with the educators concerned experiencing increased job satisfaction [6]. Educators are regarded as the facilitators of trust, in terms of creating conditions that are conducive to the development of a platform of trust [7]. Hence, the educator is in a leading position, creating challenging situations that can serve to facilitate the development of trust within the nursing education environment.

In a complex world, trust is essential to life, with it being an important component of a caring profession like nursing [8]. Jean Watson’s caring theory (1979) in [9], which refers to *caritas* processes, indicates that a helping–trusting relationship is characterised by a spirit of congruence, empathy and warmth. The engagement of a transpersonal teaching–learning experience within a caring relationship provides understanding of the perceptions involved [9]. Caring, which reflects the presence of trust, is essential to the development of professional relationships [10].

Most scholars agree on the attributes of trust [11] that may exist on multiple levels, including those of the individual, the group and the organisation. The different roles that are involved in trust can, for example, exist among educators, between educators and students, and among institutions, educators or students. Benevolence, reliability, competence, honesty and openness are important for the development and maintenance of trusting relationships in nursing schools [12]. Several studies focus on the trust that parents place in educators, the trust that exists among educators, and how the trust that is present influences the students’ achievements [11,12]. Educators’ concern regarding students’ achievements and the quality of educator and student relationships is central to teaching and learning practice [11]. Successful mentoring in nursing is based on trust, open communication and clear goal setting [13]. Nursing students reported in another study [14] that the role modelling of a mentor enhances their belief in their own abilities to succeed. Trustworthiness and the ability to guide are perceived as being important tasks of an educator in terms of nursing education [15]. In nursing education, learning is not only aimed at skills acquisition, but also at the holistic transformation of the student [16]. The development of strong, trusting relationships between educators and students improves the quality of the learning environment, with the nurse educators involved enabling their students to cultivate professional virtues. Educators should not assume that students will automatically develop ethics in class or practice [17]. Instead, educators should deliberately shape the values of nursing students, so that they align with what the nursing profession expects from them. Nursing educators should develop a professional *phronesis* in students, being practical wisdom in terms of nursing practice. The educator should demonstrate a special characteristic, such as professional *phronimos*, meaning that he/she is practically wise. Characteristics such as being honest, courageous, trustworthy and open-minded are added, as they provide insights into what moral practice nursing requires [18]. Maintaining and ensuring trust in teaching and learning are the collective responsibilities of educators in higher education institutions [19].

An inquiry into the causes of the high failure rate among nursing students, conducted by a nursing education institution in South Africa [20], revealed several concerns related to trust. High failure rates among nursing students, unapproachability of nurse educators, and negative attitudes towards student support, were some of the main findings. The knowledge of nurse educators of the content was a concern, leaving students with feelings of insecurity and fear of participating in discussion in class. Students experienced distrust in their educators and verbalised that the educators’ ‘gossip’ about them displayed favouritism and unfairness [20].

Prior studies of trust have not taken place in the context of nursing education, but, rather, between principals, educators, learners and parents within the school environment. Nursing that relies on trusting relationships should reflect how trust can be established through positive role modelling. Quality education and training are shaped with competencies such as knowledge and professional skills [19]. Hence, the problem statement reflected on aspects related to trust and trusting relationships in the nursing teaching and learning environment. The purpose of this article is to report on nurse educators’ views regarding trust and trusting relationships in the teaching and learning environment.

## Methods

### Design and settings

An explorative qualitative research design [21] was used in this study. The main aim was to explore the views of participants in relation to the phenomenon under study. The choice of this design was prompted by the limited amount of information that was available on the phenomenon of trust in nursing education and trusting relationships between educators and students. The current study was constructivist and interpretive in nature, using the constant comparative to reduce the amount of data and to develop the categories and codes involved [21].

### Study population

The target population was the overall group of persons on whom the study focused [22]. Nurse educators with theoretical and clinical experience were personally requested, recruited and selected by the researcher. The researcher visited the nursing education institutions and met with prospective participants to explain the aim of the study and the process of data collection. Those willing to participate provided their details to the researcher. The educators were employed at two accredited nursing education institutions, namely a university and a public college with three campuses situated in rural, semirural and urban areas. Using purposive sampling, educators who have had both theoretical and clinical teaching experience with an additional qualification in nursing education, were sampled. Newly appointed nurse educators with limited teaching experience were excluded from the sampling.

### Data collections and analysis

The fourteen nurse educator participants were able to contribute in-depth qualitative data on the issue of trust in nursing education [21]. Purposive sampling continued until no additional themes occurred and data saturation was evident with the refinement of categories and subcategories. Data collection was completed in 2014 after fourteen individual in-depth face-to-face interviews that were guided by a grand tour ‘question’: “Tell me your views about trust and the trust relationships in the nursing education environment that may affect teaching and learning in nursing education”. Interviews were guided by an interview schedule and conducted in the office of the participant. Data was collected through open ended questions and the interviews lasted between 45 minutes and an hour. The interviews were recorded with the permission of participants. The data was transcribed verbatim and analysed by the main researcher, supported by an independent coder, after a consensus discussion was held to reach agreement on the data analysis. Data collection and data analysis were implemented concurrently [23], following an inductive approach, with multiple truths being combined to reach the conclusion [24]. The inductive data analysis started with the first interview and lasted until data saturation. The three stages of data analysis (initial, focused and theoretical coding) were used.

Trust and trusting relationships are integral to interactions amid individuals. Current literature confirms that the findings of this study are still pertinent. The World Health Organisation [25] regards the nurse educator as someone who is expected to have the ability to nurture a relationship of reciprocal trust and respect, and exhibits interest in, and mutual respect for students. The foundational principle of adult learning highlights trust as underpinning of all relationships [26]. Therefore, cultivating trusting relationships is the cornerstone for teaching and learning. In developing trust and trusting relationships, cognitive and affective aspects are essential to facilitate trust in the nursing education environment [27]. In order for educators to facilitate trust, certain characteristics are required. The findings of this study are supported by other researchers [28] who identified three core themes namely “giving oneself”, “being competent” and “having integrity” for the development of trust in nursing education. Linked with these themes, a study [29] identified personal attributes such as caring, mutual respect, honesty and a disposition to support, as important to contribute to professional credibility and thus enhance the educator-student relationship. Expected characteristics and virtues of educators are requirements for facilitating trust and trusting relationships [30]. The themes that emerged from this study are reflected in recent literature as also mentioned here.

### Trustworthiness

Lincoln and Guba’s criteria of trustworthiness, namely credibility, transferability, confirmability and authenticity were applied [31]. Credibility refers to the truth of the participants’ views and to the prolonged engagement that took place during data collection to ensure data saturation. The criterion concerned was supported through the engagement of the researcher during the interviews, observations and audit trails. Transferability was evident due to the data saturation and the dense descriptions of data, as well as to the in-depth interviews that were conducted with the participants, which served to saturate the categories and subcategories with information. The transferability of the findings supports the criterion of dependability, ensuring that a different researcher will be able to collect similar data under similar conditions in other studies. The transferability was obtained through both memoing and an inquiry audit that was conducted by the researcher. Confirmability was demonstrated by means of the descriptions of how the conclusions were reached and the interpretations were made through the handling of the in-depth data and the dialogues of the participants. Authenticity was achieved by means of presenting the views and feelings of participants through the use of verbatim quotes.

### Ethical consideration

The three ethical principles, namely autonomy, justice and beneficence, were implemented in the current study. During the interviews, harming and exploitation of educators were excluded. The inclusion of educators were fair and the right to privacy was respected. Informed consent to participate and consent to record the interviews were obtained. Confidentiality and anonymity were carefully considered and addressed during the data collection, analysis and findings. The Health Studies’ Research Ethics Committee of the University of South Africa (Unisa) provided ethical clearance to conduct the study. Permission for the study was obtained from the Free State Department of Health, from the higher education institution concerned and from the principal of the public nursing college involved. Participation in the study was voluntary and the participants gave their informed consent in writing to the use of their data in the research.

## Results

Conclusions were drawn from the findings, with data saturation being reached after 14 in-depth interviews had been held with female nurse educators between the ages of 40 and 60 years. The participants all worked at either a nursing department at a higher education institution (i.e. a university) or a public nursing education institution (i.e. a nursing college). Three of five participants from the higher education institution held master’s degrees, whereas two held doctoral degrees. At the public nursing education institution, one of the nine participants held a master’s degree, two were busy with a master’s degree and the remaining six educators had bachelor’s degrees.

The following themes, reflecting the role of the educator as the facilitator of trust, emerged: the competencies of the educator, the setting of standards and the maintaining of consistency, professional credibility and the values of trusting relationships (see Table 1).

**Table 1:** The Educator as facilitator of trust.

Themes	Categories
Competencies of the educator	Expert knowledge
	The teaching knowledge and skills of the educator
	The ability to integrate theory with practice
	Academic support and clinical accompaniment
Setting of standards and the maintaining of consistency	Setting of standards in the teaching and learning environment
	The maintenance of consistency
Professional credibility	Attributes of trustworthiness
	Professional image
	Professional values and behaviour
Values of trusting relationships	The value of trust in the educator
	Effects of a lack of trust in the educator

### Theme 1: Competencies of the educator

In line with educators facilitating trust through their competency, the participants indicated that their competencies included their expectation that the knowledge obtained should not be restricted to the content of a subject, but that it should be extended to include teaching skills and clinical knowledge. The participants in the study considered their basic and expert knowledge, as expected by their students, to enable the development of trust in their competency as educators. Dynamic, informed, well-prepared and updated educators demonstrate expert knowledge. The updating of theoretical and practical knowledge and its alignment with current nursing practices are a requirement that they need to fulfil. The participants experienced the fact that their students often tested their competencies to verify for themselves whether or not they were informed about current practices. A participant mentioned:

“They must trust that you have their interests at heart. They must trust you. You must know your subject and demonstrate that knowledge to them. You must also demonstrate the importance of your subject area to them. So, you must sell your subject [to them], because, as soon as you do so, they will see what is important to you.”

The participants in the current study indicated the importance of their teaching knowledge and skills, in terms of the facilitation of learning. Teaching styles should accommodate students’ learning styles, so as to capture their attention and facilitate learning. The participants highlighted that the use of technology during teaching made presentations interesting and facilitated learning. The utilisation of several teaching techniques improved students’ learning abilities. One participant remarked:

“… You must make an effort with your presentations … different methods that are not the usual old standard, proper nursing education. Different methods of transferring of data, videos, slides, PowerPoint, your notes, everything.”

So as to be able to integrate theory with practice, expert theoretical knowledge and skills were required, including the ability to apply different teaching methods to facilitate the development of student trust in the educator. The participants in the study maintained that the integration of theory with practice should be linked to different subjects, so as to be able to provide a holistic learning experience. The teaching of theory and practice should be regarded as integral entities. Academic support and clinical accompaniment were to be provided to the students by the educators. The presence of the educators was found to enhance the clinical learning opportunities and motivation for the students. A participant commented as follows:

“It does help to correlate theory and practice … We need to go back to the simulation and demonstration rooms more even in our teaching … maybe I [might] say, after the lecture ‘Let’s go to the ward’, immediately, so as not to see that there is a division of block and practice placements … if I can take them to the clinical area, or to the hospital immediately to see what I taught them.”

Competent educators are seen as being able to set standards and to maintain consistency during teaching.

### Theme 2: The setting of standards and the maintaining of consistency

The participants in the current study indicated that the setting and upholding of standards in the teaching and learning environment serves to instil trust. Standardisation enhances the extent of trust that is invested in educators, with it creating a positive image of nursing. The maintenance of standards in the teaching and learning environment refers to consistency and to the expected similarities regarding educators’ assessments and procedures conducted in the theoretical and clinical environment. The element of inconsistency influences trust negatively. The participants opined that the correct standards to be upheld during the conducting of procedures were taught in class, but that the students tended to be unable to maintain the same standards in the clinical learning environment. A participant voiced her opinion as follows:

“… Students say …that: ‘We were taught differently at the college, now we are doing it differently, so … we do not trust you, because we do not see the correct things happening like we were instructed.’”

The participants in the study were concerned that, if it became apparent that different educators applied different assessment standards, the students would not trust the validity of the process. Consequently, it was felt that all educators should maintain the same standards during the assessment of students, so as to promote trust in general. Such was evident from the following remark that was made by a participant:

“That is, when they lack trust in us, …because they think [that] if you have … higher standards, they think you are the cruel one, and that one that has low standards is the good one. So, at the end of the day, we do not produce the product … you know the well-behaved, focused students, because we are having double standards.”

By maintaining consistency, the educators stated that they attempted to create a predictable environment, in which the students would be able to perform within boundaries. Policies, rules and regulations in the teaching and learning environment are set by nursing education institutions. Such policies, regulations and rules need to be applied and implemented consistently across all teaching contexts. The participants in the study indicated that policy prescriptions were to be adhered to by all the educators. One participant indicated:

“I think we need … to have discipline and we need to have consistency. Because, if you tell somebody that you want something and they do not do it, and they get away with it, then you need discipline and you need consistency.”

The participants described the inconsistencies present in clinical assessments as causing confusion, doubt and uncertainty, due to their influencing their professional credibility.

### Theme 3: Professional credibility

Professional credibility includes different attributes of trustworthiness, such as openness, honesty, reliability and caring. The participants in the study verbalised that openness and approachability were important aspects of trust between educators, and that they created a platform from which the students could confidently ask for assistance. A participant commented:

“… They [i.e. the students] lose the confidence to come to me with problems, if they think they cannot trust me. And then it causes problems for them, they do not get the help they need. They do not feel that they can go to a specific lecturer. She should also be approachable, because, sometimes, you find that they cannot trust the lecturer, because she is not approachable.”

Honesty and the quality of being ‘real’ were found by the participants in the research to create trusting relationships with the educators. Such honesty included the making of objective assessments by the educators. If the educators made mistakes, they had to admit to them, before correcting them. The ability to act fairly towards all the students was noticed, with it eliciting a positive response from the students when they found that everyone was treated equally. The participants in the study indicated that, if they admitted mistakes, doing so reflected on their reliability, with the students tending to trust those educators who were sincere. The view of a participant was:

“… You must be transparent to them. They should know what you are saying is, and should be believed, like that, not that you are actually manipulating the situation [so] that you want them to believe in that. Because, as soon as you discover what you told them was wrong, you would come back and tell them, and say that is wrong. So, they know that, now, if my lecturer says that, ‘I’ll trust her, because she’d correct it if that was not the case.’”

When students can rely on their educators, they tend to view them as being both caring and supportive. Educators should provide both academic and social support. Caring and supportive interventions by the educator should be conducted in a professional manner, and the available information should be kept confidential. A participant commented:

“If you talk about mothering somebody, it is no longer professionalism. You must be a mother, but in a professional manner. Even when you are solving a problem for students … or maybe if there is a conflict between you and a student, you do not have to be that harsh with the student. You need to be, like I indicated, that you need to adhere to the [same] standards all the time.”

Evidence of the attributes of trustworthiness helps to create a professional image for the educator. Educators must have self-knowledge, so as to be able to portray a positive professional image that is beneficial for the profession. The self-knowledge and self-trust of educators create an image of self-confidence that projects an image of trustworthiness to students. All actions of the educator should constitute those of a role model when in class and clinical areas, where interaction with the students takes place. A participant mentioned:

“If you are in class, you present yourself with dignity, this is our … your lecturer. Even the things [that] you do you should be like [those of] a role model.”

Nursing is a profession that relies on agreed-upon standards that are implemented by a professional body. Nursing as a caring profession also requires the demonstration of professional values and behaviour that cannot be standardised by a professional body, but which must emanate from the individuals themselves. Nurse educators demonstrating ethical codes, etiquette and professional values create a relationship of trust with the students. The values of the nursing school should be evident in the behaviour of educators towards their students. When educators and students share the same values of patient care, a relationship of trust is fostered between them, as one participant remarked:

“It does not help if it is documented, but not internalised, by the individual. So, in the nursing environment, I, as a lecturer, will exhibit those ethical codes [and] values, so [that they are] … visible for the student, thereby enhancing a trust relationship. I do not say it ensures it, but it should.”

Students’ trust in educators contributes to their motivation to improve, as well as to their ability to appreciate the exposure that they have to optimal learning opportunities. Having trust in the educator benefits both teaching and learning.

### Theme 4: Values implicit in trusting relationships

Trust, and a lack of trust, in the educator influence the educator’s influence on the teaching and learning environment. Having trust in the educator inspires feelings of self-worth, self-confidence and self-trust, which ultimately lead to enhanced class preparation and presentations. A trusting relationship between the educator and student tends to add value, by means of creating a relaxed learning atmosphere, in which the students are likely to be more attentive and active participants in the learning activities concerned. A participant stated:

“It is not that I will do more for that student, but it will make me feel good and motivate me to do my best in the class, and wherever I go and have anything to do with students, even in practice. [I] will be there and really try to be an example, professional and friendly … yes, I think, if she trusts me, it will motivate me to try even more. If the students trust you, it makes life much easier for you … and they are more participative in the class and cooperate. It just makes things go smoother.”

In contrast, a lack of trust in the educator can result in negative student experiences. Experiences of ignorance, of not paying attention or of not participating in learning opportunities were reported by the participants in the study. Negative student experiences tend to impact negatively on the educator, with the former tending to become defensive and guarded during student-directed learning activities. A participant who experienced a lack of trust remarked:

“The students did not trust me at all. They did not and the more I tried to tell them, but this stuff is documented, the worse it becomes. This was very bad for me as a person; I did not enjoy going to class. I needed to go and teach them, and I knew that there was not a trust relationship, so I presented formal lectures. I tried to count my words in every conversation, and [I] tried not to say anything that could lead to something [negative]. It was not nice; it really wasn’t nice.”

A trustworthy educator demonstrates various competencies, such as knowledge and teaching and clinical skills. Such competencies adhere to standards, and they are consistently recognisable. The professional image of the educator includes internal characteristics that are based on professional virtues and that are evidenced in their daily conduct. Such qualities are important in developing and maintaining a trust relationship with students.

## Discussion

### Knowledge and skills

The findings made in the present study indicate that educators should possess updated, comprehensive and expert knowledge that is subject-specific. The possession of expert knowledge and skills was found to increase the educators’ credibility and to foster the development of a relationship of trust with the students. The findings of this study revealed that educators should be able to use creative teaching methods to transfer information and knowledge, so as to be able to integrate theoretical knowledge with their clinical practice. Furthermore, the educators’ characteristics and competencies were found to be valuable in terms of the standards and trust evidenced in nursing education. The above-mentioned findings tend to support the notion that the professional knowledge of an educator should be rich and sufficiently extensive to be able to bridge theory and practice [32]. The sharing of an extensive amount of knowledge and information facilitated the students developing trust in the educators’ competencies [33]. The current study’s finding emphasised that its participants viewed educators with updated and refined clinical skills as being trustworthy, thereby pointing to a direct link between the ability to trust and the sharing of knowledge, skills, competencies and experiences [34]. Educators should acquire relevant clinical experience before assuming the role of educator [35] because trust is established when the educator shows the capability to bridge the gap between theory and practice [36]. The findings that were made in the current study reflect the notion that the educators’ updated clinical experience was able to enhance their application of effective teaching strategies, thereby enabling them to bridge the gap between theory and the clinical environment. The practice of different teaching styles and techniques and the utilising of technology, were found to tend to foster trust in the abilities of the educators concerned.

### Standards and consistency

In the present study, we concluded that maintaining standards and consistency was found to increase the level of trust present, with the findings made correlating with previous ones that revealed that educators should standardise their assessments in terms of objective assessment criteria, so as to enhance the possession of trust [19,37]. The research participants emphasised standards as being important in nursing education. Required standards, such as in the preparedness for and the presentation of lectures, should meet students’ expectations. Compiling tests and examinations must be done according to the standards of the relevant nursing school. The participants opined that differences in the educators’ application of standards resulted in a lack of trust. In previous research, congruency has been found to instil credibility [37], with the exhibiting of predictable behaviour tending to inspire trust [38]. The findings of the current study indicated that the consistency of educators, and congruency between their intentions and behaviour, support the development and maintenance of trust in nursing education. The congruency of educators, in the form of consistent professional conduct, was expected by the participants concerned. The presence of trusting relationships required proof of trustworthiness, the ability to exercise self-control and the ability to exert control over emotions, as well as predictability and consistency in behaviour.

### Professional virtues

A virtue is a moral or intellectual disposition [18], combined with practical wisdom, namely *phronesis* [39]. An educator who teaches acts as a professionally practical-wise person, *professional phronimos*, who is practically wise and sensible, while ensuring the maintenance of standards of excellence [18], knowing that possessing a good character with moral authority inspires trust [40]. The views of the participants in the current study indicate that a trusted role model with good character has competencies in terms of a specific role. Nursing etiquette and the maintenance of professional and ethical codes were regarded by the participants as being of extreme importance. The conclusion could be drawn that displaying personal and professional virtues that are in line with ethical codes reflects professional conduct, which is needed for involvement in trusting relationships. A learning environment should be conducive to the development of professional virtues and trustworthiness [18]. Benevolence, reliability, competence, honesty and openness are key to the development and maintenance of a relationship of trust [15]. The findings of the present study indicate that approachable and honest educators tend to create trusting relationships. Supportive and caring behaviour on the part of educators facilitates trust, with a caring relationship being essential to the process of learning and development [40], and trust relying on mutual support and goodwill (which are essential features of trust) [18]. The opinions of the participating educators showed that they realised that they should be mindful of their students’ academic and social problems, to be able to support their needs. Interventions conducted by educators should benefit students both academically and socially. The support provided to students created the impression that the students involved could rely on the educators. Actions of honesty, openness and approachability were found to tend to increase the amount of trust held in the educators. Consensus in teaching and assessments is known to have implications for educators’ credibility and students’ learning [40]. Theoretical and clinical assessments should be objective, fair and accurate for the development and maintenance of trust between educators and students. Objectivity is maintained by using reliable assessment methods and tools. Accordingly, the participants indicated that fairness and equal treatment served to foster trust. The possession of self-efficacy impacts on trust [41], with the degree of confidence manifested influencing the level of commitment, motivation and persistent behaviours [42]. Accordingly, the conclusion may be drawn that the amount of trust placed in educators tends to boost their self-trust and confidence. In contrast, mistrust detracts from the gains that might otherwise be made through having a positive teaching and learning environment.

### Personal attributes

Educators should have an awareness of, and should demonstrate, their personal and professional values, which should contribute to their professional credibility during the training programme for becoming a professional nurse. Their professional etiquette, as well as their professional ethics and behaviour, should be evident. Adherence to the professional code of conduct and etiquette renders the behaviour of educators predictable, with trust developing as a result. Such attributes of trustworthiness as openness, honesty, reliability, caring behaviour and goodwill should be fundamental in terms of professional conduct, so as to promote a trusting culture in nursing education. Educators should display their competencies, expert knowledge and teaching and clinical skills continuously, and they should keep abreast with the latest developments in nursing. Educators play a central role in setting standards in nursing education, with them being responsible for ensuring that standards are raised and maintained, and that needs in the theoretical and clinical environment are met. Therefore, the consistency and congruency of educators in the teaching and learning environment makes them predictable, with such predictability serving to facilitate the development and maintenance of trust and trusting relationships in nursing education.

## Conclusion

The conclusions drawn on the basis of the findings made in the present study are that educators tend to play a central role in the development of trust in nursing education. Educators represent the first contact point in the learning environment, long before students are placed in a clinical environment. Establishing trust and trusting relationships in nursing education increases the expectations that the educators involved will portray positive role modelling. As the facilitators of trust, certain competencies of the educators involved, such as expert subject knowledge and teaching competencies, support the development of trust in them. From the time of first contact between educator and students, the exertion of effort to support students’ learning opportunities serves to cultivate trust, with such effort forming a building block of the evolution of reciprocal trust. Supportive educators promote trust in the teaching and learning environment through the demonstration of approachability. Maintaining consistently high standards fosters trust in educators, as do congruency in actions, behaviours, values and fairness. Professional credibility is obtained through the demonstration of trustworthy attributes and professional conduct. An educator who facilitates teaching and learning with self-confidence is a facilitator of trust in terms of the nursing education environment. For the purpose of the current article, the views of the educators concerned, regarding their role in facilitating the development of trust and trusting relationships in the nursing education environment, were explained. A question might, nevertheless, arise regarding how the educators view the role of students within the ambit of the educator–student trust relationship. Educators’ expectations, when it comes to establishing trust in students and trusting relationships, were not discussed in the present article. However, trust and trusting relationships certainly have reciprocal expectations and responsibilities. However, the purpose of this article was to increase awareness of the nurse educators’ role in relation to establishing trust and trusting relationships. The students were seen as expecting the educators to be knowledgeable, and competent in terms of their practical and teaching skills. Professionalism and role modelling are key priorities in the trust relationships that should develop between the lecturer and student. Ensuring quality teaching and learning is key to building a foundation of trust and trusting relationships in nursing education. The development of a relationship of reputable trust during nursing education is likely to be carried over into the nursing profession, in the form of the development of trusting relationships between nurses and patients.

### Implications of the study

This study highlights the role of educators as the facilitators of trust in nursing education. Continuous awareness of factors that increase trust from students-to-educator and educator-to-students will enhance nursing education standards and role model trust and trusting in the nursing profession.

### Limitations

Few limitations in the study were identified. The study does not include the perception of registered nurses in the clinical facility. This manuscript focusses on a part of a bigger study and the results and discussion represent the role of an educator for the facilitation of trust in nursing education. Results concerning students’ views and roles for establishing trust in nursing education do not form part of this manuscript.

### Generalisation/Transferability

The knowledge generated from this study can be applied to other serving professions and the higher educational context.

### Recommendations

Before any trust or trusting relationships could be established between educators and students, self-trust of all role players is vital. Personal and professional credibility depends on the trustworthiness of each role player. Educators should foster trust through the portraying of trusting relationships with students to demonstrate building blocks of trust that may result in the development of new understandings and methods of teaching and learning in the nursing education context. These understandings may enhance the knowledge and skills of students and improve their competence in nursing.

As contribution to nursing education a substantive model was developed as an overall aim of the larger study [43]. The model for trust underlines the significance and value of trust in nursing education. Although developed from a South Africa context the model is transferable to other settings as confirmed by experts during the review process.
